# Bioengineered Platforms for Chronic Wound Infection Studies: How Can We Make Them More Human-Relevant?

**DOI:** 10.3389/fbioe.2019.00418

**Published:** 2019-12-13

**Authors:** Snehal Kadam, Shivani Nadkarni, Janhavi Lele, Savani Sakhalkar, Pratiksha Mokashi, Karishma Surendra Kaushik

**Affiliations:** ^1^Institute of Bioinformatics and Biotechnology, Savitribai Phule Pune University, Pune, India; ^2^Abasaheb Garware College, Pune, India

**Keywords:** chronic wounds, wound infection, wound models, biofilms, bioengineered platforms, *in vitro*, *ex vivo*

## Abstract

Chronic wound infections are an important cause of delayed wound healing, posing a significant healthcare burden with consequences that include hospitalization, amputation, and death. These infections most often take the form of three-dimensional biofilm communities, which are notoriously recalcitrant to antibiotics and immune clearance, contributing to the chronic wound state. In the chronic wound microenvironment, microbial biofilms interact closely with other key components, including host cellular and matrix elements, immune cells, inflammatory factors, signaling components, and mechanical cues. Intricate relationships between these contributing factors not only orchestrate the development and progression of wound infections but also influence the therapeutic outcome. Current medical treatment for chronic wound infections relies heavily on long-term usage of antibiotics; however, their efficacy and reasons for failure remain uncertain. To develop effective therapeutic approaches, it is essential to better understand the complex pathophysiology of the chronic wound infection microenvironment, including dynamic interactions between various key factors. For this, it is critical to develop bioengineered platforms or model systems that not only include key components of the chronic wound infection microenvironment but also recapitulate interactions between these factors, thereby simulating the infection state. In doing so, these platforms will enable the testing of novel therapeutics, alone and in combinations, providing insights toward composite treatment strategies. In the first section of this review, we discuss the key components and interactions in the chronic wound infection microenvironment, which would be critical to recapitulate in a bioengineered platform. In the next section, we summarize the key features and relevance of current bioengineered chronic wound infection platforms. These are categorized and discussed based on the microenvironmental components included and their ability to recapitulate the architecture, interactions, and outcomes of the infection microenvironment. While these platforms have advanced our understanding of the underlying pathophysiology of chronic wound infections and provided insights into therapeutics, they possess certain insufficiencies that limit their clinical relevance. In the final section, we propose approaches that can be incorporated into these existing model systems or developed into future platforms developed, thus enhancing their biomimetic and translational capabilities, and thereby their human-relevance.

## Chronic Wound Infections

**Figure 1:**
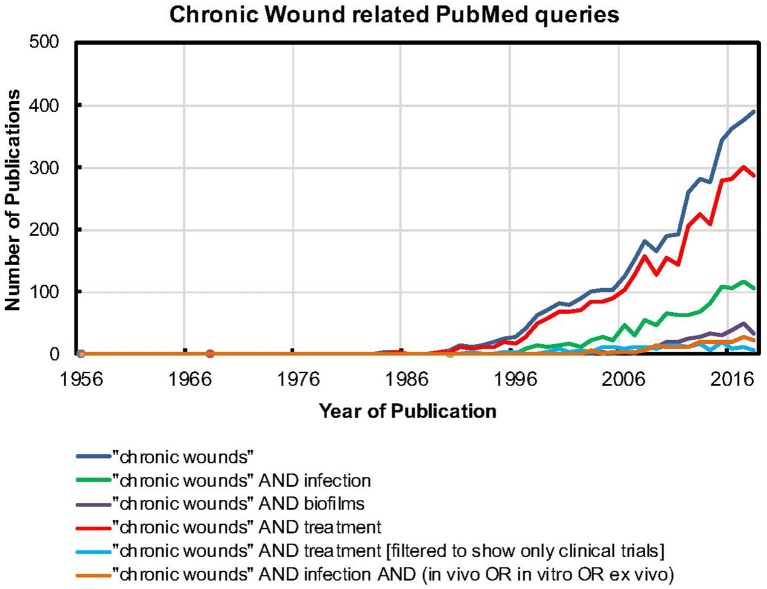
The PUBMED search tool was used to find relevant keywords in the title or main body of the article. For example, quotes were used to search for “chronic wounds” as a single term. Further, the AND operation was used to search for papers that had both “chronic wounds” as a term and the word biofilms. A similar strategy was used for all the other searches.

Cutaneous wound healing typically follows a sequential and coordinated set of processes, which includes four distinct phases of hemostasis, inflammation, proliferation, and tissue remodeling (Gurtner et al., 2008). In the proliferative-inflammatory phase, the wound bed consists of newly-laid extracellular matrix (ECM) elements, such as collagen and elastin, host cells, such as fibroblasts and keratinocytes, and is infiltrated with new blood vessels, immune cells, and proteolytic enzymes, such as matrix metalloproteinases (MMPs). This proliferative phase typically lasts for a few weeks, leading to the final stage of tissue remodeling and wound healing, without the need for significant intervention. Microbial colonization and growth, leading to wound infections, is the single-most-important cause of delayed wound healing (Church et al., 2006), resulting in a chronic, non-healing wound state. This results in a substantial healthcare burden, with millions of affected individuals, billions of dollars in costs and consequences that include a reduction in quality of life, hospitalization, amputation and even premature death (Augustin, 2013; Guest et al., 2015, 2017; Järbrink et al., 2017). In the US alone, chronic wounds affect 2% of the total population (~5.7 million individuals) and nearly 60% of these wounds are associated with microbial infections, underscoring the magnitude of the problem. Further, given the rise in diabetes, hypertension, malignancies, surgical intervention, and increasing life span, this burden is only going to increase (Guo and DiPietro, 2010). As seen in Figure 1, the number of publications on “chronic wounds” and “chronic wounds” AND treatment has steadily increased in the last three decades, likely indicating the growing magnitude of the problem and increasing focus on testing and developing therapeutic approaches. While publications with keywords “chronic wounds” AND infection and “chronic wounds” AND biofilms also show a rising trend, they are lagging behind “chronic wounds” *per se*. This possibly indicates that in recent years, infections are increasingly being studied as the leading cause of the chronic, non-healing wound state. Further, it is also being widely accepted that chronic wound infections most often take the form of biofilms, which reflects in the increasing, albeit lagging focus on “chronic wounds” AND biofilms. However, it is notable that publications on “chronic wounds” AND infection, when filtered to show model systems, be it *in vivo* or *in vitro* or *ex vivo*, are significantly lagging behind. This indicates that, in spite of a widespread understanding of infections, most often as biofilms, being a leading cause of the chronic, non-healing wound state, the development of laboratory model systems or platforms to enable their study requires greater attention. This could possibly explain why “chronic wounds” AND treatment, when filtered to show only clinical trials, is also lagging behind. A paucity of work related to developing model systems or platforms to study the chronic wound infection state very likely leads to few approaches or options available to test novel therapeutics and treatment combinations and thereby, takes these strategies toward clinical trials. Together, this data indicates a shift in the prioritization of chronic wounds from a co-morbid condition to a silent global epidemic with huge public health and economic impact. While conventional antibiotics have been the mainstay of chronic wound infection management, a range of non-conventional antimicrobial approaches, alone and in combination, have been explored (Kadam et al., 2019). To probe the potential of these therapies and develop novel treatment approaches, it is important to better understand the chronic wound infection microenvironment, including its pathophysiological features, host-microbe interactions and effects of various treatments. For this, it is important to develop model systems that recapitulate the key features and pathophysiology of the chronic wound infection microenvironment.

A large number of chronic wound infection studies have relied on *in vivo* platforms, based on live animal models. The porcine (pig) skin wound model is considered most relevant as it closely mimics the structure of human skin, providing the best representation of wound healing. However, given the cost and facilities required for large vertebrate animal care and ethical issues associated with wounding, infecting and subjecting them to experimental treatments, their applicability and availability are severely limited. Other *in vivo* models have employed rabbit, guinea pig, mouse, and rodent systems, in which following injury (or burns), wounds are infected to result in a chronic wound infection state. In general, live animal platforms offer the opportunity to mirror human wound pathophysiology, and notably enable dissection of inflammatory and immune components. However, along with cost, availability and ethical restraints, live animal testing is also limited by reproducibility, the ability to offer selective and precise control of independent factors, quantitative interpretation, and interspecies differences. On the other hand, there has been a great impetus to develop alternatives to animal research and testing, including for wound studies (Stephens et al., 2013; Caley et al., 2018). For chronic wound infections, these could potentially include bioengineered *in vitro* platforms that aim to recapitulate the key components and interactions of the infection microenvironment in a human-relevant and biomimetic manner. These *in vitro* and *ex vivo* platforms could provide a feasible and controllable, and yet biologically relevant, alternative to animal wound infection studies. Understanding the features and limitations of current bioengineered platforms and discussing approaches to make them more human-relevant would be a critical step forward.

The first section of this review outlines the key components in the chronic wound infection microenvironment, some or all of which would be important to include in a bioengineered chronic wound infection platform. In the second section, we discuss current bioengineered platforms, both *in vitro* and *ex vivo*, being employed for chronic wound infection studies. The features of these platforms have been summarized in Table 1. Finally, we propose approaches that can be incorporated into current and future platforms, toward making them more faithfully mimic the biological, biochemical, and biomechanical cues in the infection microenvironment, and thereby more human-relevant.

**Table 1:** Key features of current bioengineered platforms, *in vitro* and *ex vivo*, developed for chronic wound infection studies.

**Platform**	**Components**	**Platforms and their key features**	**References**
*In vitro*	***Microbes+Host Cells*** 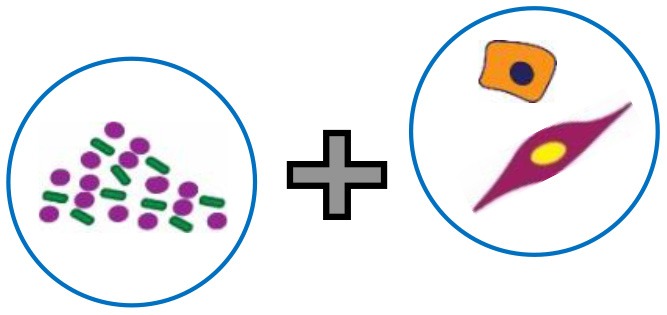	***Human Skin cells with biofilm or biofilm-conditioned media*** Study the effects of wound colonizing bacteria by co-culturing human skin cells such as keratinocytes and fibroblasts with biofilms. It recapitulates host-microbe interactions in the wound bed resulting in changes in host cell migration, proliferation, and gene expression. ***Human Skin Equivalents (HSEs)*** 3D structures that mimic human skin layers and recapitulate bacterial attachment and biofilm formation under conditions close to native architecture.	Holland et al., 2008, 2009; Charles et al., 2009; Kirker et al., 2009, 2012; Secor et al., 2011; Haisma et al., 2013; Tankersley et al., 2014; Alves et al., 2018
	***Microbes+Immune Cells*** 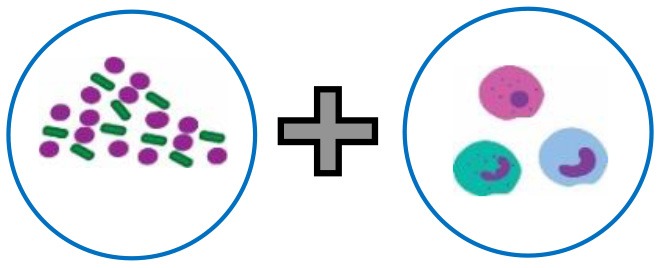	***Infection-immunity interface on a microfluidic platform*** Study interactions between the wound pathogen *S. aureus* (not specific for biofilms) and neutrophils across two compartments, enabling the study of neutrophil recruitment, migration, and engulfment.	Brackman and Coenye, 2016
	***Microbes+Extracellular Matrix*** 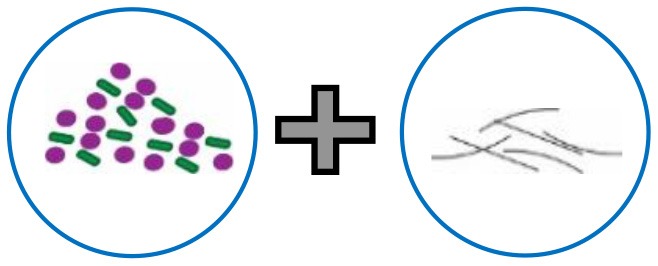	***Polymer surface coated with gel-like collagen matrix*** Study the role of matrix in biofilm formation and structure using comparisons between coated and uncoated surfaces. ***Collagen mold model with transwell inserts*** Biofilms embedded in collagen and structured as a void, recapitulating biomimetic effects such as antibiotic diffusion distance through the matrix.	Werthén et al., 2010; Price et al., 2016
	***Microbes+Wound fluid*** 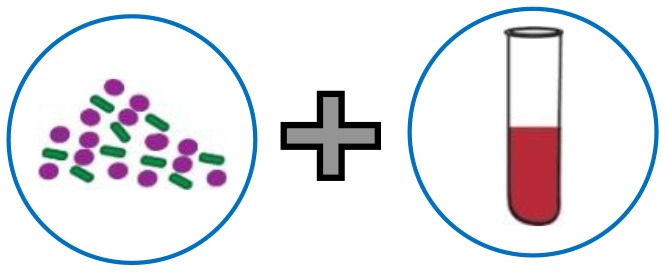	***Lubbock model (Bolton broth) and its variants*** Widely-used to mimic the wound infection state. It enables the study of biofilms and interspecies interactions and has been used to study the effects of antibiotics and other antimicrobial compounds on biofilms. ***Simulated sweat and serum media*** Enables the study of growth and biofilm formation under wound-relevant nutritional and chemical conditions.	Sun et al., 2008, 2014; Dalton et al., 2011; DeLeon et al., 2014; Dowd et al., 2014; Sojka et al., 2016
*Ex vivo*	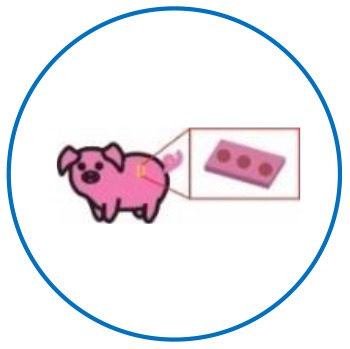	***Biological skin tissue from pigs***: A high degree of anatomic and physiological similarity to human skin and immune system. Enables the actual creation of a wound (thermal injuries, infected state). Biological tissue supports biofilm growth. Enables testing of immune parameters such as cytokine responses. Can be leveraged to test therapeutics under closely human-relevant conditions.	Steinstraesser et al., 2010; Yang et al., 2013; Thet et al., 2016
	***Porcine skin***		
	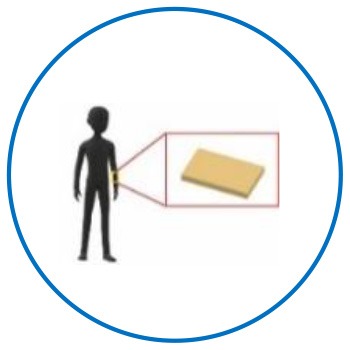	***Biological tissue from human skin:*** Can faithfully recapitulate biomimetic features of the chronic wound infection state. Demonstration of biofilm formation and critical host immune factors including cellular and cytokine responses. Can be leveraged to test therapeutics under human-relevant conditions.	Misic et al., 2014; Schaudinn et al., 2017; Ashrafi et al., 2018
	***Human skin***		

## Key Components of the Chronic Wound Infection Microenvironment

**Figure 2:**
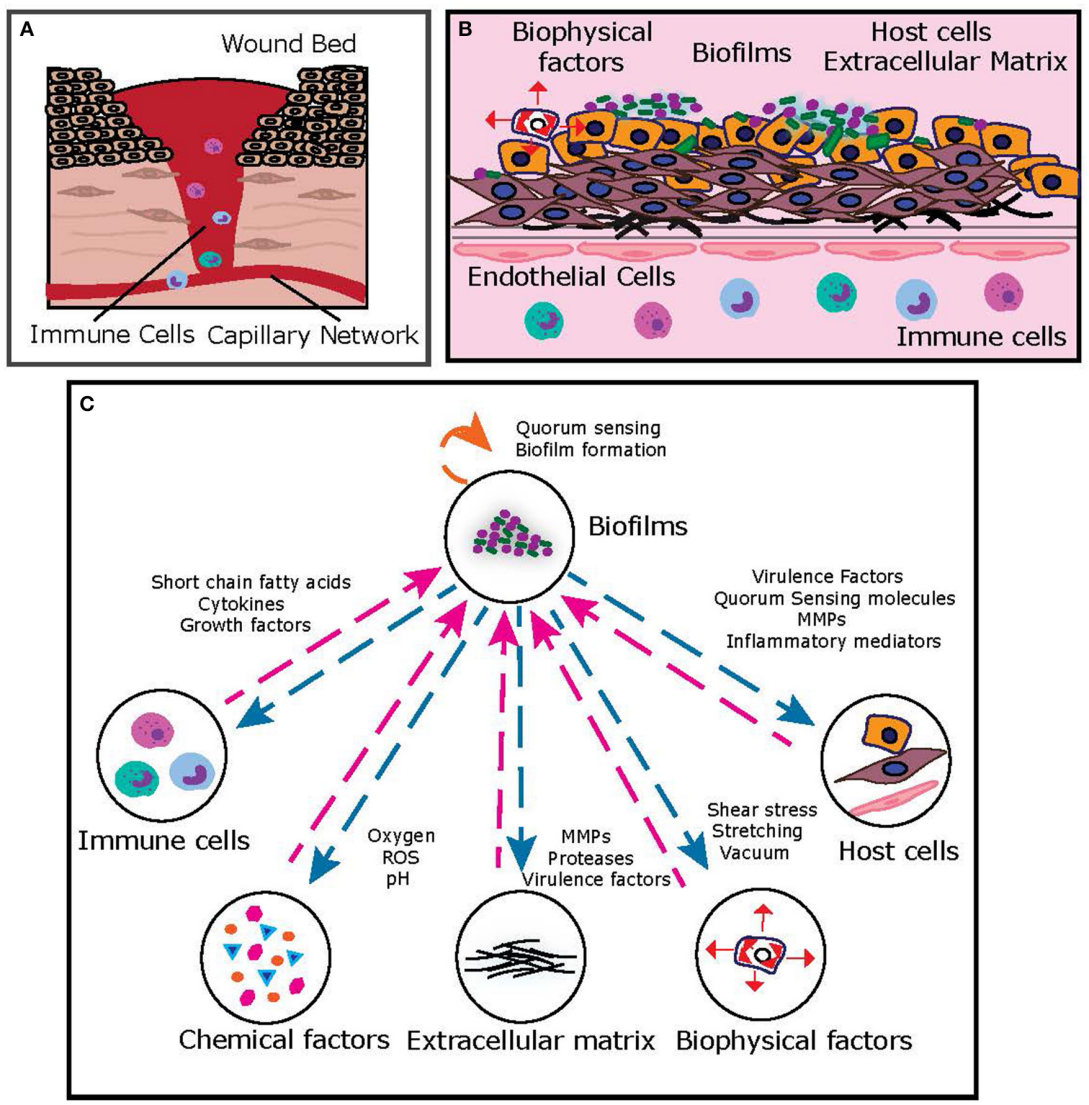
**(A)** Typical representation of the chronic wound bed microenvironment. **(B)** Key features of the chronic wound bed-capillary interface. From a bioengineering standpoint, the microenvironment can be represented by a two-compartment system, where the upper compartment consists of the “infected wound bed” with host cells, matrix and microbial biofilms and the lower compartment represents the capillary interface (endothelial cells) with immune components. **(C)** A simplified representation of key interactions between chronic wound biofilms and other key components of the chronic wound microenvironment, which can be suitably dissected on human-relevant bioengineered platform.

In the inflammatory-proliferative phase, the “wound bed” is progressively filled with granulation tissue, composed of fibroblasts and keratinocytes in an extracellular matrix of collagen, elastin, and fibronectin (Figure 2). Formation of new blood vessels (angiogenesis), results in a wound bed-capillary interface (Tonnesen et al., 2000), across which host cellular and matrix elements (fibroblasts, keratinocytes, collagen) communicate with immune cells (neutrophils, monocytes, macrophages) via a regulatory network of inflammatory cytokines, growth factors, and matrix metalloproteinases (MMPs) (Ravanti and Kähäri, 2000; Barrientos et al., 2008). Microbial colonization and proliferation in the wound bed most often takes the form of biofilms, highly structured bacterial communities embedded in a self-produced extracellular polymeric substance, which are notoriously recalcitrant to antibiotics and immune clearance, resulting in a chronic, non-healing wound state (Bowler et al., 2001). Microbial components [bacteria, toxins, virulence factors, metabolites, short-chain fatty acids (SCFAs), quorum signals] communicate with host and immune cells, further contributing to the complexity of signaling in the dynamic chronic wound infection state (Rumbaugh et al., 2015). In addition, mechanical forces (such as shear flow and compression), regulate host and microbial elements to influence this network (Zhou et al., 2013). As evident, the chronic wound infection milieu consists of several key players that interact with each other and the environment via complex and dynamic signaling networks, resulting in a pathophysiological infected state.

### Microbial Components

It is well-established that microbes in chronic wound infections typically exist in a biofilm state (Attinger and Wolcott, 2012; Zhao et al., 2013; Clinton and Carter, 2015). In a study involving clinical wounds, biofilms were demonstrated in nearly 60% of chronic wounds, representing an almost 10-fold higher association as compared to acute wounds (James et al., 2008; Malone et al., 2017). The presence and persistence of biofilms in chronic wounds affects a range of host cellular, inflammatory and innate immune elements, such as neutrophils, macrophages, cytokines, and matrix-degrading metalloproteinases (Grice and Segre, 2012; Zhao et al., 2013; Dhall et al., 2014).

Microbial wound communities are most often polymicrobial, with two or more species of microbes occupying the infection site (Kirketerp-Møller et al., 2008). These include both aerobic and anaerobic species of bacteria, encompassing a wide range of pathogens. Of the aerobes and facultative bacteria, *Staphylococcus aureus, Pseudomonas aeruginosa*, and β-hemolytic Streptococci remain the primary causes of chronic wound infections (Bowler et al., 2001). While estimation of the anaerobic burden of chronic wound infections remains a challenge, owing to the lack of appropriate culture and isolation practices, they are believed to form a significant proportion of the microbial population (Sun et al., 2014; Omar et al., 2017). Other bacteria found in chronic wound infections include *Enterococcus* spp*, Klebsiella pneumoniae, Acinetobacter baumanii, and Enterobacter* spp. (ESKAPE pathogens), coagulase-negative Staphylococci, and *Proteus* species (Bowler et al., 2001). While the clinical relevance of fungi in chronic wound infections has been understudied, they constitute a significant component of the wound microbial burden, and several endogenous fungi, including *Candida, Curvularia*, and *Malasezzia* have been implicated in chronic wound infections (Kalan and Grice, 2018). Interactions between different bacterial species, between bacterial and fungal species, and microbial components and microenvironmental factors are known to affect the progression and outcome of wound infections. For example, co-infection with *P. aeruginosa* and *S. aureus* is associated with higher inflammatory responses, increased antimicrobial tolerance and contributes to the chronic, non-healing state. In another study, in an early-stage wound biofilm model, where *P. aeruginosa* and *S. aureus* were co-cultured on human keratinocytes, *S. aureus* mediated a significant increase in the attachment and aggregation of *P. aeruginosa* (Alves, P. M. et al., 2018). On the other hand, in biopsies from wounds later in the course of infection, *P. aeruginosa* and *S. aureus* are observed to occupy different niches in the wound microenvironment, with *P. aeruginosa* aggregates located deeper in the wound, as compared to the more superficially located *S. aureus* (Fazli et al., 2009). The deep *P. aeruginosa* aggregates produce virulence factors that destroy infiltrating neutrophils, which also results in a constant cycle of recruitment of neutrophils and persistent inflammatory state. This indicates that this niche partitioning that occurs over time is likely beneficial for maintaining a stable, persistent infection state (Alves, P. M. et al., 2018). In an *inter-kingdom* wound infection model, *C. albicans* and *Citrobacter freundii* were shown to assemble (Kalan et al., 2016) into structured, three-dimensional biofilms, where *C. albicans* provided a scaffold for *C. freundii* to attach and proliferate. Microscopic evidence of the interaction suggested that the fungi themselves were critical to the construction and establishment of the biofilm. Similar inter-kingdom interactions were demonstrated in a tripartite, three-dimensional biofilm model of *C. albicans, Staphylococcus aureus*, and *P. aeruginosa*, using a hydrogel scaffold to mimic a wound surface. Here, *C. albicans* grew predominantly as yeast cells, as opposed to hyphae, and co-aggregated with bacterial cells (Townsend et al., 2016).

### Host Cellular and Matrix Components

The chronic wound bed consists of fibroblasts and keratinocytes embedded in an extracellular matrix (ECM) of collagen, fibrin, fibronectin, elastin, proteoglycans, and glycosaminoglycans, all of which are critical for wound repair (Tracy et al., 2016). While ECM components are known to provide a scaffold for cellular support, they are also known to influence cell survival, proliferation and function (Tracy et al., 2016). In chronic wound infections, biofilms are found as aggregated colonies scattered in the upper layers of the wound, as well as embedded in the collagen network (Schaudinn et al., 2017). The presence and persistence of biofilms in chronic wounds has been shown to affect keratinocyte and fibroblast functions, such as inflammation, chemotaxis, and the release of growth factors, cytokines, and MMPs (Zhao et al., 2013; Tankersley et al., 2014). For example, biofilms are shown to induce a characteristic low-grade inflammatory and secretory response, which correlates with impaired keratinocyte migration and altered cell morphology (Zhao et al., 2013; Jeffery Marano et al., 2015). In another study, conditioned medium from methicillin-resistant *S. aureus* cultures, differentially affected the production of a range of inflammatory and growth factors, including matrix metalloproteinases (Kirker et al., 2012). The cell and matrix-rich wound bed is closely approximated with a robust network of newly-sprouted capillaries (as a result of angiogenesis), constituting the dynamic wound bed-capillary interface (DiPietro, 2016). Across this interface, a range of chemical, biological and immune factors, such as inflammatory mediators, communicate with and influence microenvironmental factors (Zhao et al., 2013). Angiogenesis is critical to enable wound healing, and biofilm formation and soluble biofilm factors from wound pathogens have been demonstrated to affect capillary sprouting (Ward et al., 2015). This results in poor vascularization, which further contributes to the chronic wound state via hypoxia and reduced micronutrient delivery.

### Immune Factors

Following wounding, microbial colonization or infection rapidly induce the host innate immune response (Grice and Segre, 2012; Strbo et al., 2014), in which neutrophils are the first inflammatory cells at the wound site (Wilgus et al., 2013). Neutrophils phagocytose microbes in the wound bed while secreting a plethora of cytokines, proteolytic enzymes (such as matrix metalloproteinases, elastase, cathepsin G) and antimicrobial peptides (Dale et al., 2008; Wilgus et al., 2013; Florez-Sampedro et al., 2018). Other immune cells, such as monocytes (which differentiate to macrophages) also play a role in phagocytosis while producing a range of cytokines and growth factors, including TNFα and interleukins (Dale et al., 2008; Florez-Sampedro et al., 2018). Further, resident cells, such as keratinocytes produce antimicrobial peptides, recruit immune cells, and induce the production of cytokines, further contributing to the inflammatory cascade (Richmond and Harris, 2014; Albanesi et al., 2018). In infected chronic wounds, a state of unresolved inflammation persists, which is amplified by the presence of pathogenic microbes in the form of biofilms (Zhao et al., 2013). This leads to an increased influx of neutrophils, macrophages and inflammatory mediators (Wilgus et al., 2013; Landén et al., 2016; Krzyszczyk et al., 2018). For example, the infected chronic wound bed is characterized by an excess production of matrix metalloproteinases (MMPs), mainly by macrophages, and a decreased production of the natural tissue inhibitors of MMPs called TIMPS (Zielins et al., 2014; Ayuk et al., 2016). This prolonged state of inflammation leads to the degradation of newly-formed ECM and production of abnormal ECM forms (chronic granulation tissue). Degraded matrix elements act as chemotactic peptides, attracting more inflammatory neutrophils into the wound, resulting in a non-healing state of perpetual chronic inflammation (Adair-Kirk and Senior, 2008; Zhao et al., 2013).

### Nutrient and Chemical Factors

In addition to complex biological interactions, the chronic wound infection bed is subject to nutrient factors and chemical gradients, which influence several critical functions. Chronic wound fluid is an important factor in the wound milieu (Trengove et al., 1996; Cutting, 2003; Widgerow, 2011; Manuela et al., 2017), reflecting the processes and functions that contribute to and perpetuate the chronic wound state. In addition to a unique signature of proteins, growth factors and proinflammatory cytokines, chronic wound fluid is known to contain high levels of matrix metalloproteinases (MMPs), which impact a range of functions related to the chronic wound state (Wysocki et al., 1993; Jindatanmanusan et al., 2018). For example, increased levels of the enzymes MMP-2 and MMP-9 have been found to correlate with the severity of chronic wounds (Grzela et al., 2016). These MMPs are known to digest ECM components, and excessive levels are likely to impair normal tissue repair processes. On the other hand, the antibiotic doxycycline has been shown to inhibit the activity of certain MMPs (Stechmiller et al., 2010), raising the possibility that the chronic wound milieu can be leveraged as a target and tool for effective therapeutics.

Among chemical factors, sufficient oxygen supply is an essential component of successful wound healing; several biochemical and cellular processes involved in would healing are oxygen-dependent, including the production of reactive oxygen species, microbial killing, deposition and remodeling of the ECM, inflammatory signaling cascades (Hall and Mah, 2017; Mashruwala et al., 2017). A typical characteristic of chronic wounds is sustained inadequate oxygenation of the wound bed, owing partly to impaired vascularity (angiogenesis), which increases the diffusion distance for oxygen (Castilla et al., 2012; James et al., 2016). This results in a heterogeneous redox environment, which not only influences the structure and function of key wound healing components but also impacts the response to therapy (Gottrup, 2004; Sen, 2009; Castilla et al., 2012). In the context of chronic wound infections, tissue hypoxia promotes the growth of facultative microbes, which in turn consume oxygen and further lowers its availability (Bowler et al., 2001). While the exact mechanisms underlining the impact of oxygen on chronic wound biofilms remains to be explored, it remains plausible that lowered oxygen tension and redox potential will favor the proliferation of polymicrobial biofilms in the wound bed (Cramer, 2014). In addition, anoxic or hypoxic conditions have been shown to reduce the efficacies of a range of antibiotics, resulting in reduced biofilm susceptibility (Schaible et al., 2012; Antoniazzi et al., 2016; Gupta et al., 2016). Further contributing to this redox state are excess levels of reactive oxygen species (ROS), which are considered to be a major cause of delayed wound healing. These ROS molecules include hydrogen peroxide, hydroxyl radicals, and superoxide anions, and result from an increased initial influx and oxidative burst of inflammatory cells, such as neutrophils and macrophages in chronic wounds. Increased ROS is linked to activation of proteolytic enzymes, endothelial damage and delay in re-epithelialization, leading to a perpetual cycle of tissue damage and chronic inflammation (Dunnill et al., 2017).

Another important chemical microenvironmental factor in chronic wound infections is pH. The pH of the wound bed is known to affect key factors involved in wound healing, such as angiogenesis, keratinocyte and fibroblast migration and signaling, matrix metalloproteinase activity, and microbial proliferation. An acidic environment is considered to favor wound healing; notably, chronic wound infections are typically characterized by an alkaline pH (Rumbaugh et al., 2015). This alkaline pH results from an interplay of several factors, including inadequate oxygenation and the presence of bacterial infection. On the other hand, alkaline pH is known to regulate bacterial virulence and biofilm formation (Percival et al., 2014; Rippke et al., 2018). Further, the efficacy of several therapeutic approaches, such as probiotics, enzymatic debridement and antibiotics is dependent on wound pH (Schneider et al., 2007; Rumbaugh et al., 2015). For example, alkaline pH has been shown to enhance the activity of aminoglycosides, such as tobramycin (Kaushik et al., 2015). While this synergistic effect was noted with planktonic *P. aeruginosa* cells, the effect was observed to be antagonistic with for the biofilm form (Kaushik et al., 2016). Overall, recapitulating the chemical microenvironment of chronic wounds will aid the development of management strategies that can improve wound infection outcomes under these conditions.

### Biophysical Factors

Wounds are affected by a variety of biophysical forces, which include mechanical tension, compression, stretching, shear stress, and osmotic forces (Barnes et al., 2017). Following injury, the response of various wound components to these mechanical forces is crucial for the subsequent healing process. In the chronic wound infection microenvironment, the biological behavior of biofilms, host and immune cells and extracellular matrix elements are influenced by mechanical cues. The effects of mechanical forces, particularly shear stress, on the structure, formation and function of microbial biofilms, have drawn increasing interest. For example, in *P. aeruginosa* biofilms, shear stress affects biochemical signaling pathways that affect the production of the matrix exopolysaccharides and induces the transition from planktonic to biofilm lifestyle (Rodesney et al., 2017). In addition, biomechanical forces critically influence the breakup, dispersal and seeding of new biofilms, determining whether a biofilm spreads over a surface or strengthens itself (Thomen et al., 2017). Biophysical forces, such as tension, cyclical stretching and vacuum are also well-known to affect host cellular elements, such as fibroblasts and keratinocytes (Yano et al., 2004; Gupta and Grande-Allen, 2006; Reichelt, 2007; Agha et al., 2011). These forces modulate intracellular signaling pathways in fibroblasts and keratinocytes, leading to increased cellular proliferation, migration, and production of extracellular matrix. Similarly, the influx and phagocytic activity of immune cells, such as neutrophils and monocytes, is influenced by biophysical forces (Ostrowski et al., 2016). For example, reperfusion of wounds (following ischemia-reperfusion injury) results in an overabundance of inflammatory cells (including neutrophils) and cytokines (Kalogeris et al., 2012; Zhao et al., 2015). Excessive neutrophil infiltration sets up a cycle of chronic inflammation and is a biological marker for non-healing wounds.

### Features of an Ideal Model System for Chronic Wound Infections

An ideal chronic wound infection model system should be capable of not only incorporating all key components that constitute the chronic wound bed but also recapitulating the structure, dimensionality, architecture and functioning of the infection microenvironment. In chronic infected wounds, these typical pathophysiological features include: (1) Presence of microbial elements in the form of biofilms. (2) Poorly responding host cells, such as fibroblasts, keratinocytes. (3) A state of perpetual wound inflammation, mediated by an increased influx of immune cells, such as neutrophils. (4) Deficient and defective extracellular matrix production (ECM). (5) Failure of re-epithelialization due to lack of sufficient ECM and scaffold components (Demling, 2006; Kadam et al., 2019).

While biofilms models of chronic wound infections have been developed (Ganesh et al., 2015; Bahamondez-Canas et al., 2019), to recapitulate the chronic wound infection microenvironment, in addition to microbial elements, the bioengineered platform should include host cellular and matrix components (including immune cells and endothelial cells), relevant nutrient conditions and biomechanical forces. To recreate the wound bed, host cells, such as keratinocytes and fibroblasts would need to proliferate simultaneously and in a stratified fashion, where keratinocytes overlay fibroblasts. Further, these host cellular elements should be capable of producing and proliferating in the presence of matrix components (such as collagen, elastin, fibrinogen), thereby constituting the wound granulation tissue. To represent the capillary interface, this granulation tissue should include endothelial cells, preferably as capillary networks, in intricate proximity and communication (via relevant growth factors) with other key components. In order to mimic the chronic wound infection state, it would be imperative to include relevant microbial pathogens, growing as multicellular, three-dimensional biofilm aggregates in close association with host cellular elements. To leverage this platform for host-microbe interactions, it would be critical to include immune cells, such as neutrophils and macrophages, with the ability to respond to and influence host cells and microbial biofilms. Finally, the chronic wound infection platform should be subject to biophysical forces, such as stretching, compression, and shear stress, known to impact the viability and functioning of various cellular and microbial elements.

## Bioengineered Platforms for Chronic Wound Infection Studies

Given the presence of multiple components and interactions in the chronic wound infection state, it is rather idealistic to expect a bioengineered platform to recreate this complex multidimensionality. However, there has been substantial work toward developing *in vitro* and *ex vivo* platforms that recapitulate the infected wound state, notably the presence of microbial pathogens in the presence of key wound bed components.

### *In vitro* Systems

#### Matrix-Based Platforms

The extracellular matrix of the chronic wound bed is a complex structure of structural proteins, such as collagen, elastin, glycosaminoglycans, proteoglycans, fibronectin, and laminin. Given that the wound bed extracellular matrix plays an important role in wound healing and infection outcome (Tracy et al., 2016), it would be very relevant and important for wound infection models to incorporate these matrix elements. *In vitro* models that incorporate specific matrix elements, focus largely on collagen as the main component (Werthén et al., 2010; Price et al., 2016). In one such example, a polymerized gel-like cross-linked matrix made of rat tail Type I collagen was coated on a polymer surface and employed as a substrate for biofilm attachment and growth (Werthén et al., 2010). The substrate was prepared by mixing collagen with fetal calf serum and peptone water, thereby simulating the protein-rich wound microenvironment. When grown in presence of the collagen matrix, *S. aureus* showed enhanced biofilm formation as compared to an uncoated surface. On the other hand, presence of the collagen substrate did not alter *P. aeruginosa* biofilms, as compared with biofilms grown on uncoated surfaces. The presence of serum, however, impacted biofilms formed by both species, whereby it inhibited *S. aureus* biofilm formation and enhanced *P. aeruginosa* surface attachment. Further, the formed biofilms showed the presence of bacterial aggregates very similar to aggregates observed in chronic wound tissues infected with *P. aeruginosa*.

In another example of a matrix-based chronic wound infection model, type I collagen was polymerized around a mold in a transwell insert, allowing a void to be formed, thereby mimicking an ulcer (Price et al., 2016). The void was inoculated with *S. aureus* or *P. aeruginosa*, which formed biofilms embedded in the collagen matrix. The biofilms were then exposed to antibiotic-loaded calcium sulfate beads, which were added to the void. This treatment successfully inhibited bacterial growth, as seen by a reduction in viable cells. Notably, this platform revealed that a higher concentration of antibiotic was required to remove biofilms from the collagen model as compared to a biofilm grown on a plastic peg, underscoring the importance of recapitulating the physical structure of the wound bed. The presence of collagen allowed the bacteria to form more resistant biofilms, probably related to an increased diffusion distance for the antibiotic via dense matrix components.

#### Host Cell-Based Platforms

Human cell lines are an important component that make *in vitro* models more human-relevant. A few studies investigate the effects of wound colonizing bacteria by co-culturing host cells that constitute the wound bed, such as keratinocytes and fibroblasts, with biofilms or in biofilm-conditioned media (Kirker et al., 2009, 2012; Alves, P. M. et al., 2018). These models typically use transwell inserts to grow biofilms, and subsequently, the biofilm inserts or biofilm conditioned media is added to host cell cultures. A similar cell culture-based platform revealed that secreted products from *S. aureus* biofilms resulted in keratinocyte apoptosis, reduced migration and proliferation and prevented *in vitro* scratch wound closure (Kirker et al., 2009). Similar model systems have also been useful in elucidating gene expression and differential cytokine production in keratinocytes exposed to biofilm conditioned media (Secor et al., 2011; Tankersley et al., 2014). A similar cell culture-based platform has also been used to study the effects of *S. aureus* biofilms with dermal fibroblasts (Kirker et al., 2012), and revealed impaired migration and increased cell death with both, media conditioned with biofilms and planktonic cells. While these are important steps toward studying long-term host-microbe interactions relevant to chronic wound infections, it would be important to extend these platforms to include other wound pathogens as well as multi-species biofilms.

In addition to these host cell line-based platforms, *in vitro* bioengineered cell-culture based 3D models, also known as Human Skin Equivalents (HSEs), have also been employed to develop wound infection models. These HSEs are made by culturing fibroblasts in a collagen matrix, after which keratinocytes are seeded onto the fibroblasts. These HSEs are then inoculated with bacteria to study bacterial attachment and biofilm formation. Studies with laboratory-developed and commercial HSEs demonstrate successful biofilm formation with *S. aureus* and *P. aeruginosa* as well as gene expression changes in the HSEs due to the infection (Holland et al., 2008, 2009; Charles et al., 2009; Haisma et al., 2013). HSEs can either be constructed using detailed protocols (Carlson et al., 2008), or commercially available HSE constructs can be bought (Horch, 2009). While some commercial products have been developed to model relevant human biology for research^1^, others have been developed as skin substitutes (Horch, 2009) to assist wound healing (Streit and Braathen, 2000; Veves et al., 2001).

#### Wound Fluid-Based Models

Wound fluid represents the composite wound milieu and reflects critical processes and functions that contributing to the chronic wound state (Trengove et al., 1996; Cutting, 2003; Widgerow, 2011; Manuela et al., 2017). The composition and characteristics of wound fluid, including nutrient and biochemical factors, impact microbial growth and proliferation in the wound bed. Chronic wound infection models have been developed that aim to mimic the wound fluid milieu by using combinations of serum, red blood cells, plasma, and brain heart infusion media.

The Lubbock model uses Bolton broth (a combination of heparinized bovine plasma and horse red blood cells) to grow multispecies biofilms, with the broth acting as the growth media, and the pipette tip used for inoculation (which is left inside the inoculation tube) acting as the surface for biofilm formation. This model and its variants have been used to test antibiotics and antimicrobial compounds, study inter-species interactions within the biofilm and grow biofilms for subsequent *in vivo* studies (Sun et al., 2008, 2014; Dalton et al., 2011; DeLeon et al., 2014; Dowd et al., 2014; Sojka et al., 2016).

Another interesting study compared the growth of wound pathogens methicillin-resistant *S. aureus* and *P. aeruginosa* in the presence of commensal skin bacteria, in a simulated sweat and serum media (Oates and McBain, 2016). In the planktonic state, both wound pathogens had a higher growth rate in the simulated fluids as compared to commensal bacteria. On the other hand, in a solid substrate foam-based model, prior colonization with commensal bacteria reduced colonization and integration of the wound pathogens into the foam-based model. This likely indicates the complexity of interspecies interactions and competition between pathogens and commensal bacteria under *in vivo* like conditions.

#### Infection-Immunity Interface Platforms

Neutrophils play an important role in the clearance of biofilms in wounds and wound healing (Wilgus et al., 2013; De Oliveira et al., 2016), yet incorporating immune elements into bioengineered *in vitro* models remains a challenge. One of the major reasons is that neutrophils have a short lifespan (Summers et al., 2010) (with a half-life of 6–8 h), which makes them difficult to culture under laboratory conditions. Therefore, experimental setups require freshly isolated neutrophils from human subjects (Kuhns et al., 2015). In spite of these technical limitations, interactions between neutrophils and biofilms at different sites, such as on dental and ocular surfaces, have been well-explored (Hirschfeld, 2014; Oveisi et al., 2019; Papayannopoulos, 2019; Thanabalasuriar et al., 2019), which could serve as a template for developing immune-infection interface studies for chronic wound biofilms.

A recent study used a microfluidic device to study neutrophil migration and the killing of microbes by freshly isolated neutrophils (Ellett et al., 2019). The microfluidic device had an inner chamber where *S. aureus* cells were introduced and an outer chamber where neutrophils were introduced and allowed to migrate into the inner chambers, thereby recapitulating an infection-immunity interface. Additionally, the use of pH specific reporters allowed visualization of phagosome acidification after engulfment of bacteria within neutrophils. Neutrophils from healthy donors demonstrated migration toward the bacteria and suppression of bacterial growth, whereas neutrophils from patients with sepsis under intensive care showed lower recruitment and suppression. While this work explores the infection-immunity interface for a wound relevant pathogen, it does not specifically incorporate or study the biofilm state. However, these *in vitro* platforms highlight the potential to dissect interactions between chronic wound biofilms and immune cells, and thereby enable unprecedented insights into the wound infection microenvironment.

### *Ex vivo* Systems

*Ex vivo* platforms of chronic wound infections are, to a large extent, capable of accounting for the native architecture and multidimensionality of the infection microenvironment, including a range of relevant features, such as host tissue components and immune signaling (Nunan et al., 2014; Brackman and Coenye, 2016). While human skin would serve as an ideal model system to study chronic wound infections, ethical and clinical considerations preclude its extensive use. This prompts the need for animal model systems, in which animal skin is excised (*ex vivo*), followed by wounding and introduction of relevant microbial components, to simulate an infected wound state. Porcine skin models are considered to be the best representation of human wound infections, given that they share a high degree of anatomic and physiological similarity to human skin (Sullivan et al., 2001; Middelkoop et al., 2004; Seaton et al., 2015; Jensen et al., 2017). Further, the porcine immune system is highly similar to the human immune system, making it well-suited to study host-microbial interactions (Ganesh et al., 2015; Jensen et al., 2017).

#### *Ex vivo* Porcine Skin Models

Alves, D. R. et al. (2018) employed the use of an *ex vivo* porcine skin model as a substrate to study *S. aureus* biofilm formation and virulence gene expression in thermally induced wounds. In this model, a burn wound array device (BWAD) is used to create partial-thickness thermal injuries, following which the skin is excised and employed as a substrate for microbial inoculation. The chronic wounds generated were able to support biofilm formation and provided a suitable model system to study metabolic activity, gene expression and virulence of *S. aureus* biofilm infection. The model was further leveraged to evaluate the ability of a bacteriophage to inhibit *S. aureus* biofilm formation. While this model did not examine polymicrobial biofilm formation, other *ex vivo* porcine chronic wound infection models have not only recapitulated the biofilm growth of numerous clinically relevant species, including co-pathogens *P. aeruginosa* and *S. aureus* (Thet et al., 2016) but have also displayed the characteristic increased tolerance to antibiotics (Yang et al., 2013). Importantly, the *ex vivo* burn wound array device (BWAD) model reported biofilm formation only for a short duration of 4 days and was not extended to study host immune response factors.

#### *Ex vivo* Human Skin Models

This critical host immune component has been elicited and demonstrated in an *ex vivo* human skin based wound infection model (Steinstraesser et al., 2010; Schaudinn et al., 2017). In recent work (Schaudinn et al., 2017), wounds were induced by intradermally injecting *P. aeruginosa*, Following infection, bacteria were observed to spatially-segregate, where the upper wound layers showed dense bacterial communities in the native collagen network, and scattered bacterial aggregates in the deep wound bed, closely resembling bacterial aggregates seen in chronic wounds (Kirketerp-Møller et al., 2008). This is important to note, as it indicates that this *ex vivo* model system was capable of recapitulating histological findings from human chronic wound biopsy specimens. Importantly, this bacterial infection was capable of invoking an immune response in the *ex vivo* skin, as demonstrated by an increase in selective interleukin levels. Notably, levels of IL-1α and IL-1β were increased early in infection, on stimulation of keratinocytes, fibroblasts, macrophages and dendritic cells. On the other hand, IL-6 and IL-8 levels were found to be below detection limits in the infected skin. This is surprising, given that the levels of IL-6 and IL-8 should be elevated during a bacterial infection, owing to their release from fibroblasts and macrophages. This indicates the need to fine-tune the *ex vivo* platform to recapitulate host-microbe interactions and the inflammatory state possibly more faithfully.

Recently, a human *ex vivo* cutaneous wound model for bacterial biofilms was developed to study the profile of volatile organic compounds (VOCs) produced by the metabolic activity of biofilm bacteria (Ashrafi et al., 2018). Using a comparison between *in vitro* biofilm growth on a polyester substrate and human *ex vivo* wound biofilms, identification and relative abundances of distinct VOC profiles were obtained for *S. aureus, P. aeruginosa* and *S. pyogenes*. Unique VOC profiles were not only obtained for biofilms of the different bacterial species, but the signature profiles also varied based on the model platform. While these findings hold potential clinical applicability for the efficient and non-invasive diagnosis of wound infections, they also underline the importance of developing model systems with a high degree of human relevance and clinical translatability.

## How can We Make Bioengineered Chronic Wound Infection Platforms More Human-Relevant?

As previously described, current bioengineered chronic wound infection platforms are either simple, *in vitro* platforms with various combinations of host cellular elements, immune cells, wound matrix, mono-or multi-species bacterial biofilms, and mechanical factors, or *ex vivo* biological tissue systems. While these systems incorporate several relevant aspects of the complex chronic wound infection microenvironment, there are inherent limitations in their biological translatability. This could be related to the architecture of the platform, the ability to simultaneously propagate multiple cellular elements, sustain host-microbial co-culture for a relevant duration, provide optimum nutrient conditions, or incorporate biophysical forces. In addition to incorporating these aspects, a bioengineered platform should also provide selective and precise control of different factors, enable the dissection of specific host-microbe interactions, and possess a high degree of reproducibility and tractability. This would lead to an unprecedented understanding of the chronic wound infection microenvironment, and thereby be more relevant for subsequent clinical, translational and therapeutic studies.

There are several strategies that, if incorporated into current or future bioengineered chronic wound infection platforms, can make them more closely mimic the chronic wound infection state.

### Polymicrobial Biofilm Communities

Given that microbial communities in chronic wounds are almost always polymicrobial, it is imperative that platforms engineer multi-species biofilm consortia, with relevant wound pathogens (Bowler et al., 2001; Misic et al., 2014). Further, given the growing significance of inter-kingdom signaling incorporating relevant fungal elements (Kalan and Grice, 2018) could more accurately reflect microbial burden, diversity and interactions in the infected wound bed.

### Nutrition and Growth Conditions

The wound bed is bathed in wound fluid, consisting of a complex milieu of nutrients, growth factors, small molecules, and immunomodulatory components (Cutting, 2003). While the constituents of wound fluid will vary based on the stage of chronicity, presence of microbial elements, and host status (Hanson et al., 2005), recapitulating key characteristics, such as growth of biofilms, host cell culture, and host-microbe co-culture in the presence of relevant nutritional and growth media is important. For this, developing and employing simulant media that mimic the wound fluid milieu, as opposed to standard laboratory culture media, would be a valuable first step.

### Cellular and Matrix Components

Establishing chronic wound infection models that recapitulate the host-microbe interface at the wound bed, by incorporating host cells, such as fibroblasts and keratinocytes, would enable the dissection of these interactions and how they impact the infected wound physiology. A few wound infection models have been developed employing fibroblast and keratinocyte cell lines, as well as using human skin equivalents (HSEs) and pre-manufactured skin layers (Carlson et al., 2008; Holland et al., 2008, 2009; Charles et al., 2009; Haisma et al., 2013; Kirker and James, 2017). In a recent study, chronic wound fibroblasts were isolated from patients with non-healing leg ulcers and proposed as a model cell line. Chronic wound fibroblasts are known to have characteristics patterns of gene expression, migration, morphology and proliferative capacity, as compared with normal skin fibroblasts. Incorporating cell lines, such as these chronic wound fibroblasts into wound infection platforms would be another step toward recapitulating relevant host characteristics. On the other hand, incorporating matrix elements is relatively simpler. Matrix components in the wound bed play a critical role in wound infection process including biofilm formation and development. Current and future chronic wound infection models can aim to include several key matrix elements in addition to collagen, such as fibrinogen (Chen et al., 2014) and elastin, and leverage them as a scaffold to grow biofilms. However, to successfully recreate the wound infection microenvironment, it would also be imperative to establish and maintain stable biofilm growth in these cellular and matrix platforms, which can prove to be challenging.

### Biophysical Factors

Future bioengineered platforms could also be built to be amenable to apply and incorporate biophysical forces relevant to the infected wound bed, such as shear stress, compression, and stretching forces (Farahani and Kloth, 2008; Lu and Kassab, 2011; Wong et al., 2012; Korzendorfer and Hettrick, 2014). This is particularly relevant to understand these effects on biofilm structure, formation and function, as well as inform approaches to remove and prevent biofilms. This work has been attempted with model systems designed on microfluidic devices or 3D platforms (Conant et al., 2010; Conde et al., 2013; Perestrelo et al., 2015; Brann et al., 2017; Srinivasan et al., 2017; Go et al., 2018; Liu et al., 2018) that incorporate flow of media and lateral forces, and variations of this approach could be devised for wound infection relevant studies. A recent study developed a microfluidic lab-on-a-chip method to induce mechanical circular “wounds” in confluent cell layers (Sticker et al., 2017), allowing the study of highly reproducible wounds. These platforms have allowed co-culture of various mammalian cells in neighboring wells/troughs (keratinocytes, fibroblasts, endothelial cells) to study their effects on wound healing (An et al., 2015), and could be extended to study the infected state of the wound. In this context, it would also be important to measure the various biophysical parameters exerted in these platforms quantitatively, and possibly compare the forces exerted with what is known to be present in the wound bed (Farahani and Kloth, 2008; Lu and Kassab, 2011; Wong et al., 2012; Korzendorfer and Hettrick, 2014).

### Immune Cells and Factors

Immune and inflammatory components, such as neutrophils, monocytes (which differentiate to macrophages), cytokines, growth factors and antimicrobial peptides finely calibrate the pathogenic state of the infected wound bed. It would be relevant for infected wound model systems to employ immune components to establish an infection-immunity interface. A relatively simple way to do this would be to incorporate immune signaling factors, such as cytokines and antimicrobial peptides, at or near the biofilm, in the nutrient media or across a semi-permeable barrier (Gopalakrishnan et al., 2015). A more complex approach is to introduce freshly-isolated neutrophils across a semi-permeable membrane or within the biofilm matrix (Oveisi et al., 2019).

## Conclusions

A range of chronic wound infection platforms have been developed that have significantly advanced our understanding of the basic pathophysiology of chronic wound infections and provided insights into therapeutics. While they possess certain insufficiencies that limit their clinical relevance and translational potential, there are several strategies that can be incorporated into them to make them more closely simulate the infected wound microenvironment and thereby more human-relevant. Going forward, this should be a major focus area, likely to be best achieved by intersections between various fields, such as bioengineering, tissue engineering, microbiology (particularly the biofilm field), immunology, biological physics and clinical areas, such as wound care, infectious diseases, antibiotic resistance, and alternative antimicrobial approaches.

## Author Contributions

SK, SN, JL, SS, PM, and KK devised the framework of the manuscript and wrote the manuscript. SK and KK made the figures and tables.

### Conflict of Interest

The authors declare that the research was conducted in the absence of any commercial or financial relationships that could be construed as a potential conflict of interest.

## Footnotes

^1^MatTek Corporation|Biotechnology Company. Available online at: https://www.mattek.com/
