# Corrigendum: Bioengineered Platforms for Chronic Wound Infection Studies: How Can We Make Them More Human-Relevant?

**DOI:** 10.3389/fbioe.2019.00449

**Published:** 2020-02-04

**Authors:** Snehal Kadam, Shivani Nadkarni, Janhavi Lele, Savani Sakhalkar, Pratiksha Mokashi, Karishma Surendra Kaushik

**Affiliations:** ^1^Institute of Bioinformatics and Biotechnology, Savitribai Phule Pune University, Pune, India; ^2^Abasaheb Garware College, Pune, India

**Keywords:** chronic wounds, wound infection, wound models, biofilms, bioengineered platforms, *in vitro*, *ex vivo*

In the original article, there was a mistake in the legend for **Figure 2** as published. The legend incorrectly cites a reference for Figure 2(A) as “modified from.” The figure was in fact made by the authors. The correct legend appears below.

Figure 2. **(A)** Typical representation of the chronic wound bed microenvironment. **(B)** Key features of the chronic wound bed-capillary interface. From a bioengineering standpoint, the microenvironment can be represented by a two-compartment system, where the upper compartment consists of the “infected wound bed” with host cells, matrix and microbial biofilms and the lower compartment represents the capillary interface (endothelial cells) with immune components. **(C)** A simplified representation of key interactions between chronic wound biofilms and other key components of the chronic wound microenvironment, which can be suitably dissected on human-relevant bioengineered platform.

Additionally, there was a mistake in Table 1 as published. The last row of the table had an incorrect placement of the figures. The corrected Table 1 appears below.

**Table 1:** Key features of current bioengineered platforms, *in vitro* and *ex vivo*, developed for chronic wound infection studies.

**Platform**	**Components**	**Platforms and their key features**	**References**
*In vitro*	***Microbes+Host Cells*** 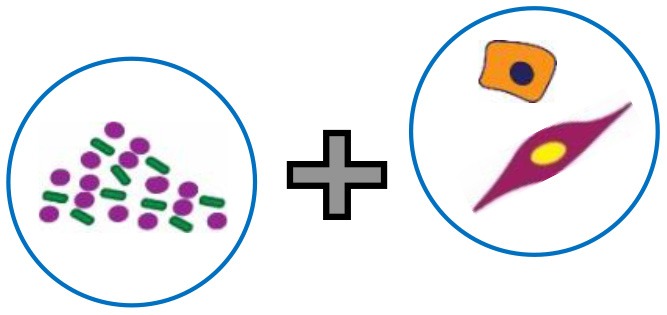	***Human Skin cells with biofilm or biofilm-conditioned media*** Study the effects of wound colonizing bacteria by co-culturing human skin cells such as keratinocytes and fibroblasts with biofilms. It recapitulates host-microbe interactions in the wound bed resulting in changes in host cell migration, proliferation, and gene expression. ***Human Skin Equivalents (HSEs)*** 3D structures that mimic human skin layers and recapitulate bacterial attachment and biofilm formation under conditions close to native architecture.	Holland et al., 2008, 2009; Charles et al., 2009; Kirker et al., 2009, 2012; Secor et al., 2011; Haisma et al., 2013; Tankersley et al., 2014; Alves et al., 2018
	***Microbes+Immune Cells*** 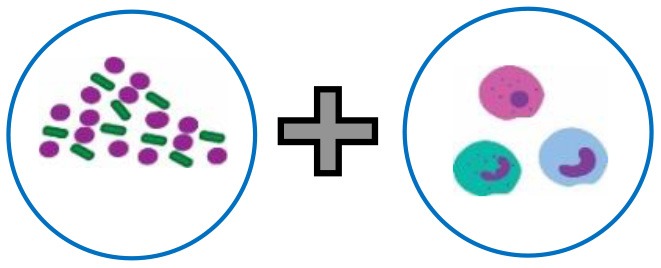	***Infection-immunity interface on a microfluidic platform*** Study interactions between the wound pathogen *S. aureus* (not specific for biofilms) and neutrophils across two compartments, enabling the study of neutrophil recruitment, migration, and engulfment.	Brackman and Coenye, 2016
	***Microbes+Extracellular Matrix*** 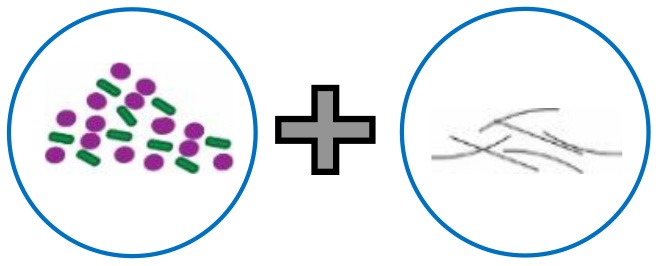	***Polymer surface coated with gel-like collagen matrix*** Study the role of matrix in biofilm formation and structure using comparisons between coated and uncoated surfaces. ***Collagen mold model with transwell inserts*** Biofilms embedded in collagen and structured as a void, recapitulating biomimetic effects such as antibiotic diffusion distance through the matrix.	Werthén et al., 2010; Price et al., 2016
	***Microbes+Wound fluid*** 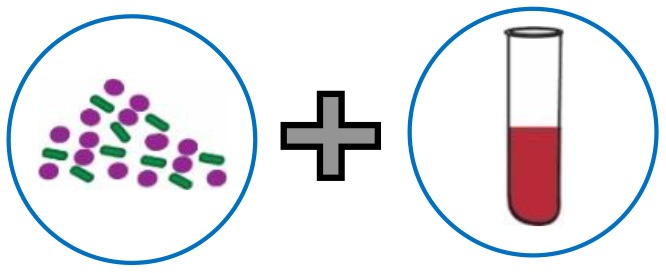	***Lubbock model (Bolton broth) and its variants*** Widely-used to mimic the wound infection state. It enables the study of biofilms and interspecies interactions and has been used to study the effects of antibiotics and other antimicrobial compounds on biofilms. ***Simulated sweat and serum media*** Enables the study of growth and biofilm formation under wound-relevant nutritional and chemical conditions.	Sun et al., 2008, 2014; Dalton et al., 2011; DeLeon et al., 2014; Dowd et al., 2014; Sojka et al., 2016
*Ex vivo*	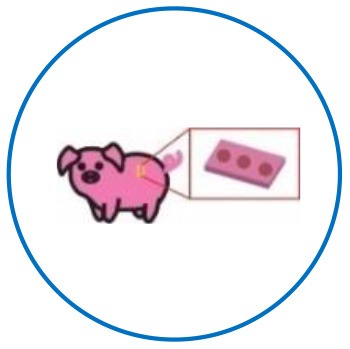	***Biological skin tissue from pigs***: A high degree of anatomic and physiological similarity to human skin and immune system. Enables the actual creation of a wound (thermal injuries, infected state). Biological tissue supports biofilm growth. Enables testing of immune parameters such as cytokine responses. Can be leveraged to test therapeutics under closely human-relevant conditions.	Steinstraesser et al., 2010; Yang et al., 2013; Thet et al., 2016
	***Porcine skin***		
	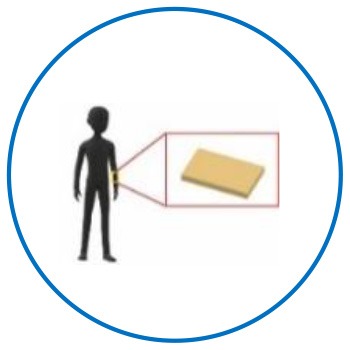	***Biological tissue from human skin:*** Can faithfully recapitulate biomimetic features of the chronic wound infection state. Demonstration of biofilm formation and critical host immune factors including cellular and cytokine responses. Can be leveraged to test therapeutics under human-relevant conditions.	Misic et al., 2014; Schaudinn et al., 2017; Ashrafi et al., 2018
	***Human skin***		

The authors apologize for these errors and state that this does not change the scientific conclusions of the article in any way. The original article has been updated.
